# Effect of an exercise-based cardiac rehabilitation program on quality of life of patients with chronic Chagas cardiomyopathy: results from the PEACH randomized clinical trial

**DOI:** 10.1038/s41598-024-58776-3

**Published:** 2024-04-08

**Authors:** Marcelo Carvalho Vieira, Fernanda de Souza Nogueira Sardinha Mendes, Paula Simplício da Silva, Gilberto Marcelo Sperandio da Silva, Flavia Mazzoli-Rocha, Andrea Silvestre de Sousa, Roberto Magalhães Saraiva, Marcelo Teixeira de Holanda, Daniel Arthur Barata Kasal, Henrique Silveira Costa, Juliana Pereira Borges, Michel Silva Reis, Luiz Fernando Rodrigues Junior, Alejandro Marcel Hasslocher-Moreno, Pedro Emmanuel Alvarenga Americano do Brasil, Mauro Felippe Felix Mediano

**Affiliations:** 1 Evandro Chagas National Institute of Infectious Disease, Oswaldo Cruz Foundation, Rio de Janeiro, RJ Brazil; 2 Center for Cardiology and Exercise, Aloysio de Castro State Institute of Cardiology, Rio de Janeiro, RJ Brazil; 3 Department of Research and Education, National Institute of Cardiology, Rio de Janeiro, RJ Brazil; 4 Internal Medicine Department, State University of Rio de Janeiro, Rio de Janeiro, RJ Brazil; 5 Department of Physical Therapy, Federal University of Jequitinhonha and Mucuri Valleys, Diamantina, MG Brazil; 6 Laboratory of Physical Activity and Health Promotion, University of Rio de Janeiro State, Rio de Janeiro, RJ Brazil; 7 Faculty of Physical Therapy, School of Medicine, Federal University of Rio de Janeiro, Rio de Janeiro, RJ Brazil; 8 Department of Physiological Sciences, Federal University of the State of Rio de Janeiro, Rio de Janeiro, RJ Brazil

**Keywords:** Cardiac rehabilitation, Chagas cardiomyopathy, Neglected diseases, Exercise, Heart failure, Quality of life, Cardiomyopathies, Heart failure, Quality of life, Rehabilitation

## Abstract

To investigate the effect of an exercise-based cardiac rehabilitation program on the quality of life (QoL) of patients with chronic Chagas cardiomyopathy (CCC). PEACH study was a single-center, superiority randomized clinical trial of exercise training versus no exercise (control). The sample comprised Chagas disease patients with CCC, left ventricular ejection fraction < 45%, without or with HF symptoms (CCC stages B2 or C, respectively). QoL was assessed at baseline, after three months, and at the end of six months of follow-up using the SF-36 questionnaire. Patients randomized for the exercise group (n = 15) performed exercise training (aerobic, strength and stretching exercises) for 60 min, three times a week, during six months. Patients in the control group (n = 15) were not provided with a formal exercise prescription. Both groups received identical nutritional and pharmaceutical counseling during the study. Longitudinal analysis of the effects of exercise training on QoL, considering the interaction term (group × time) to estimate the rate of changes between groups in the outcomes (represented as beta coefficient), was performed using linear mixed models. Models were fitted adjusting for each respective baseline QoL value. There were significant improvements in physical functioning (β =  + 10.7; *p* = 0.02), role limitations due to physical problems (β =  + 25.0; *p* = 0.01), and social functioning (β =  + 19.2; *p* < 0.01) scales during the first three months in the exercise compared to the control group. No significant differences were observed between groups after six months. Exercise-based cardiac rehabilitation provided short-term improvements in the physical and mental aspects of QoL of patients with CCC.

Trial registration: ClinicalTrials.gov Identifier: NCT02517632; August 7, 2015.

## Introduction

Chagas disease (CD) is a parasitic infection caused by the protozoan *Trypanosoma cruzi*, which can progress into chronic Chagas cardiomyopathy (CCC) in approximately 30% of chronically infected patients^1^. CCC is characterized by arrhythmias, thromboembolism, and heart failure (HF), which may cause fatigue, dyspnea, and gradual decline in functional capacity, ultimately decreasing patients’ quality of life (QoL)^2^.

Patients with CCC typically experience lower QoL compared to healthy individuals^3^, those with the indeterminate form of CD^4,5^, and individuals with HF from other etiologies^5–7^. QoL can reflect the patient’s perception of a disease’s functional impact on different aspects of life^3^, and has recently emerged as an important health construct associated with hospitalization and mortality^8,9^. In patients with CCC, QoL has proven to be useful in screening for cardiac dysfunction^10^ and predicting adverse cardiovascular events^11^.

Exercise-based cardiac rehabilitation (CR) holds potential value in improving the QoL of patients with different heart diseases^12–14^. However, little evidence is available addressing the influence of CR on QoL in patients with CCC, as these patients are typically not included in clinical trials^15^. To the best of our knowledge, only three articles^16–18^ have examined the impact of exercise on QoL in patients with CCC, yielding divergent results and some methodological limitations. Lima et al. encountered measurement interpretation issues and included patients with preserved functional class^16^, while Mediano et al. and Mediano et al. used a single-group design with a small sample size^17,18^. Therefore, the impact of CR on QoL in CCC patients remains uncertain. Evaluating and comprehending these aspects is of paramount importance, considering the clinical complexities of the disease. Understanding QoL allows healthcare providers to tailor treatment plans and interventions to address not only the medical aspects of a condition, but also its impact on patients' overall well-being, resulting in a more effective and comprehensive care.

Thus, we aimed to investigate the effect of an exercise-based CR program on the QoL of patients with CCC. We hypothesized that exercise training would improve both the physical and mental aspects of QoL in these patients.

## Methods

### Study design

The complete description and the main results for clinical and functional variables of the PEACH study (“Exercise Program in Chagas Heart Disease” in Portuguese) have been previously published^19,20^. In short, PEACH study was a single-center, superiority randomized parallel-group clinical trial of exercise training versus no exercise training (control), conducted from March 2015 to January 2017 at the Evandro Chagas National Institute of Infectious Diseases (INI) of the Oswaldo Cruz Foundation (Fiocruz). Individuals followed at INI were sequentially recruited to participate in the study. The sample comprised CD patients (confirmed by two distinct serological tests) of both sexes, older than 18 years, diagnosed with CCC, left ventricular ejection fraction (LVEF) < 45%, without or with HF symptoms (CCC stages B2 or C, respectively), New York Heart Association (NYHA) functional class I or II during three months before study enrollment, clinically stable and under optimal medical therapy according to HF guidelines over the prior six weeks before study enrollment. Exclusion criteria were the presence of major comorbidities or limitations that could preclude exercise training, pregnancy, unavailability to attend exercise sessions 3 times a week, practice of regular exercise training at baseline (> 1 week) in the three months prior to the study, smoking, or evidence of associated non-CCC.

Sealed envelopes filled with a computer-generated sequence were used to randomly allocate the eligible patients between the two groups in a 1:1 ratio using WinPepi software. The sequence was generated in blocks and stratified according to CCC stages (B2 and C) by a single researcher who was not involved in the recruitment.

Sample size calculation for the present study considered a significant mean difference between groups of 42.98 in the SF-36 role limitations due to physical problems scale, with a standard deviation of 39.43 points for the control and 34.32 points for the intervention group^21^. Assuming α = 0.05 and β = 0.20, 26 participants were required (13 in the intervention group and 13 in the control group).

### Measurements

Sociodemographic (age, sex, schooling, and self-reported race), anthropometric, clinical, cardiac function, and maximal progressive cardiopulmonary exercise test (CPET) variables of the eligible patients were obtained during the initial assessment.

Schooling included the years of formal study, stratified into two categories (< 9 years and ≥ 9 years). Self-reported race was recategorized as white and non-white (including black, mulatto, indigenous, and yellow). Anthropometric evaluation consisted of measurements of height and weight, according to Lohman et al.^22^. Body mass index (BMI) was calculated as the ratio of weight (kg) to height squared (m^2^) and classified according to the World Health Organization definition^23^. Clinical variables, such as stage of CCC, NYHA functional class, presence of electrocardiogram abnormalities (ventricular premature beats, sustained and non-sustained ventricular tachycardia, right branch block, left anterior hemiblock, non-specific ventricular repolarization changes, atrial fibrillation, first-degree atrioventricular block, and second-degree atrioventricular block), and medications, were obtained from medical records. Systolic cardiac function was determined by means of left ventricular ejection fraction (LVEF) using the modified Simpson’s rule^24^. Maximal symptom-limited CPET was performed on a treadmill (Inbramed, Porto Alegre, RS, Brazil) with a ramp protocol and active recovery, using a VO_2000_ gas analyzer (MedGraphics, St. Paul, MN, USA) connected to a computerized Ergo PC Elite system (Micromed, Brasília, DF, Brazil).

QoL was assessed at baseline, after three months and at the end of follow-up (six months) using the Portuguese version of the Medical Outcomes Study 36-Item Short-form of Health Survey (SF-36) questionnaire^25–27^. This instrument has good internal consistency, test–retest reliability, and construct validity in Brazilian populations across different age groups and health conditions, including individuals with CCC^11,27–32^. SF-36 is a generic multidimensional instrument consisting of 36 questions, referring to the four weeks prior to the interview and divided into eight different scales: physical functioning, role limitations due to physical problems, bodily pain, general health perceptions, vitality, social functioning, role limitations due to emotional problems and mental health. These scales define two summary scores: physical component summary (PCS) and mental component summary (MCS). Both PCS and MCS have contributions from all eight scales, but with higher weights for the first four scales in the PCS and for the last four scales in the MCS. The final score ranges from zero (worse QoL) to 100 (best QoL)^25,26^.

### Intervention

Patients randomized for the exercise group performed physical exercise sessions for 60 min, three times a week, for six months. Each session consisted of 30 min of aerobic exercise on a treadmill or cycle ergometer, 20 min of strength training comprising two sets of 12 repetitions for the major muscle groups (sit-ups, push-ups, and pull-ups), and 10 min of stretching exercises. Aerobic exercise intensity was set according to the heart rate at anaerobic threshold obtained during the CPET (90–100% of heart rate at anaerobic threshold in the first month of exercise training and 100–110% of heart rate at anaerobic threshold thereafter)^33^. The anaerobic threshold values used to determine the target heart rate were derived from the baseline cardiopulmonary exercise tests for training sessions conducted between baseline and the 3rd month, and from the 3-month cardiopulmonary exercise test for training sessions between the 3rd and 6th months. For those patients in which anaerobic threshold was not identified during the CPET (n = 13; 43%), training intensity was prescribed according to the Hellerstein formula [HR = (102 + maximum metabolic equivalents achieved)/1.41)]^34^, in which the target heart rate ranged from 70% of maximum heart rate obtained in the CPET to the Hellerstein’s formula percentage in the first month, and from the Hellerstein’s formula percentage to 85% of maximum heart rate in the following months. Borg scale was used as an adjuvant of exercise intensity prescription (targeting 2–4 in CR10 Borg scale). The intensity of exercise was controlled by an exercise physiologist that supervised all exercise sessions. All exercise sessions were center-based and carried out in the morning, in an indoor environment with controlled temperature and under a multidisciplinary supervision (including an exercise physiologist and physician). No guidance was given to participants for home-based exercises (outside of the center-based program). Patients in the control group were not provided with a formal exercise prescription.

During the study, patients from both groups underwent monthly appointments with their cardiologist, based on the recommendations of the Brazilian Consensus on CD^35^. In addition, both groups received identical nutritional and pharmaceutical counseling during the study. Nutritional counseling consisted of general orientation about healthy eating habits, such as reducing the consumption of saturated fatty acids and increasing the intake of poly- and monounsaturated fatty acids, vitamins, and high-fiber carbohydrates. Reductions in sodium consumption and water intake were also stimulated for those patients with HF. Pharmaceutical care consisted of guiding about medication usage, drug dosage, and compliance, added to the monthly distribution of personalized packages, with the pills organized by the time and days that should be taken, according to medical prescription^19^.

### Data analysis

Descriptive analysis consisted of mean and standard deviation for continuous variables and number of observations and percentage for categorical variables. The longitudinal analysis of the effects of exercise-based CR on QoL was performed using mixed linear models. This approach enable us to assess the interaction term (group x time) to estimate the rate of changes between groups in the outcomes. Models were fitted adjusting for each respective baseline QoL value. All participants were considered in the statistical analysis, regardless of compliance or loss to follow-up, characterizing an intention-to-treat analysis. Line graphs were constructed to visually illustrate the crude trajectories of QoL scales during the follow-up in each group.

The Research Electronic Data Capture (REDCap) web application was used for data management and the data analysis was conducted using Stata 13.0. Statistical significance was set at *p* ≤ 0.05 for all analyses.

### Ethical considerations

All participants received information about the goals and procedures of the study and voluntarily agreed to participate by means of signing a written informed consent. The study was performed in accordance to the resolution 466/2012 of the Brazilian National Council of Health and was approved by the Institutional Research Ethics Committee (CAAE: 38038914.6.0000.5262) in February, 2015. The clinical trial was registered at ClinicalTrials.gov (NCT02517632).

## Results

**Figure 1:**
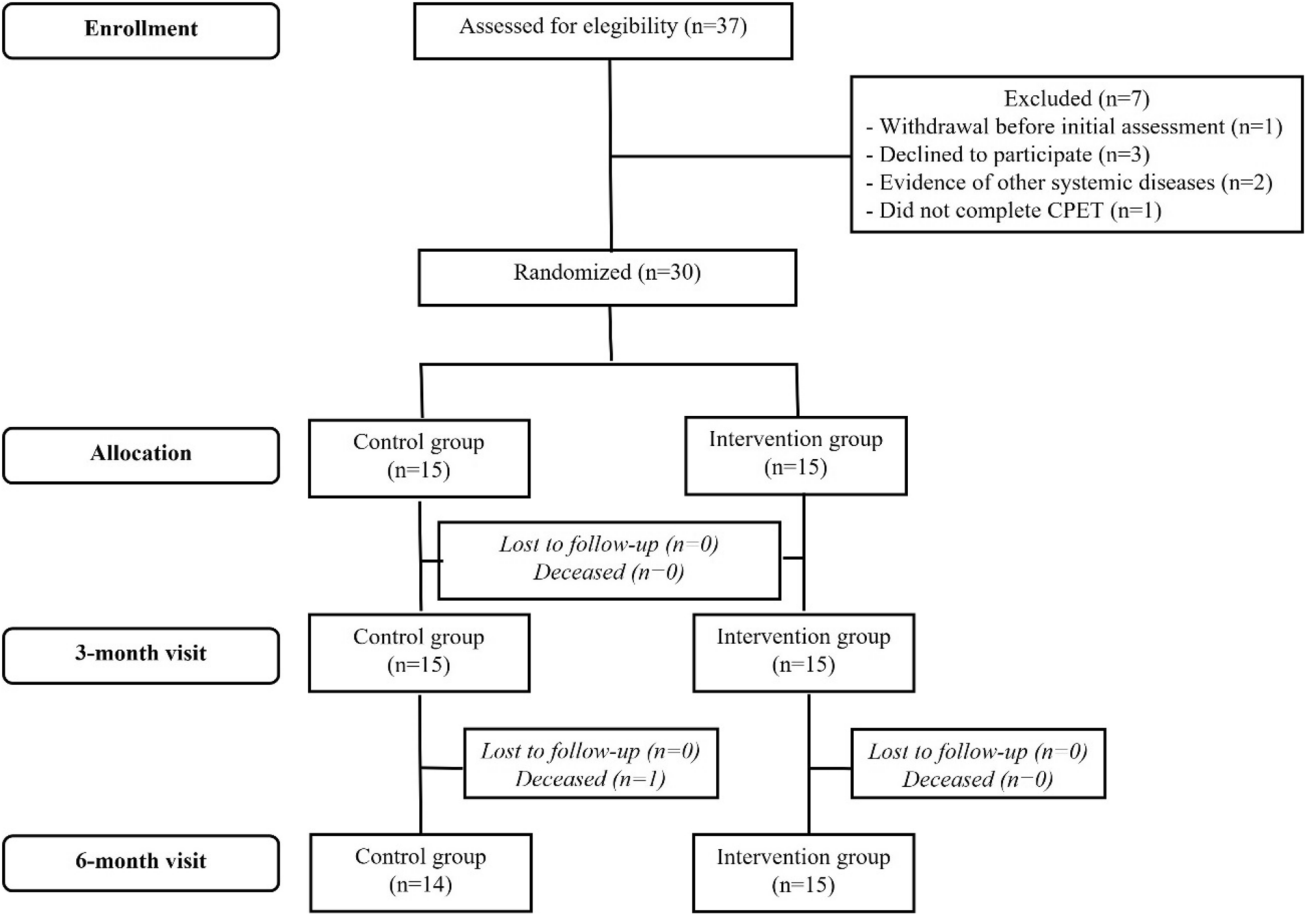
Flow chart of screening, randomization, and follow-up of participants included in the study.

A flow chart of participant inclusion and follow-up is depicted in Fig. 1. Losses to follow-up during the six months were restricted to one participant in the control group that died between 3- and 6- month visits from non-CCC related cause. Exercise group achieved 80% of attendance to training sessions during the first three months and an overall compliance of 74% at the end of six months of follow-up.

### Sample characteristics

Baseline characteristics of the patients included in each arm are shown in Table 1. The overall mean age was 59.8 (± 10.0) years, most were men (66.7%), with the majority presenting stage C of CCC (73.3%). In relation to baseline QoL assessments, patients presented lower scores in the physical scales compared to the mental scales, with the overall mean summary scores equal to 43.0 (± 9.8) for PCS and 53.0 (± 11.7) for MCS.

**Table 1:** Baseline characteristics of participants included in the study (n = 30).

Variable	Control (n = 15)	Exercise (n = 15)
Age (years)	60.7 (10.6)	57.9 (9.5)
Sex		
Women	6 (40.0%)	4 (26.7%)
Men	9 (60.0%)	11 (73.3%)
Schooling		
< 9 years	13 (86.7%)	12 (80.0%)
≥ 9 years	2 (13.3%)	3 (20.0%)
Race		
White	4 (26.7%)	8 (53.3%)
Non-white	11 (73.3%)	7 (46.7%)
Clinical form of CCC		
B2 (without heart failure)	4 (26.7%)	4 (26.7%)
C (with heart failure)	11 (73.3%)	11 (73.3%)
LVEF (%)	30.9 (7.0)	32.3 (8.7)
VO_2_ peak (ml kg^−1^ min^−1^)	15.4 (6.3)	17.6 (4.7)
BMI (kg/m^2^)	25.6 (4.3)	25.1 (6.2)
Hypertension	1 (6.7%)	0 (0.0%)
Diabetes mellitus	2 (13.3%)	3 (20.0%)
Dyslipidemia	4 (26.7%)	5 (33.3%)
Stroke	0 (0.0%)	5 (33.3%)
Electrocardiogram abnormalities	12 (80%)	10 (66.7%)
Cardiac device	9 (60.0%)	5 (33.3%)
Medications^a^		
Beta-blocker	14 (93.3%)	15 (100%)
ACEI or ARB	14 (93.3%)	14 (93.3%)
Diuretics	10 (66.7%)	12 (80.0%)
Aldosterone antagonist	8 (53.3%)	7 (46.7%)
Amiodarone	2 (13.3%)	4 (26.7%)
Anticoagulants	7 (46.7%)	7 (46.7%)
SF-36 QoL scales and summaries		
Physical functioning	60.7 (26.2)	71.0 (26.5)
Role limitations due to physical problems	58.3 (46.0)	61.7 (46.2)
Bodily pain	59.8 (19.9)	87.1 (19.0)
General health perceptions	53.6 (18.1)	70.8 (23.2)
Physical component summary	39.5 (9.3)	46.6 (9.3)
Vitality	60.0 (29.4)	73.7 (22.8)
Social functioning	85.2 (21.9)	81.0 (25.8)
Role limitations due to emotional problems	64.4 (42.7)	66.7 (45.4)
Mental health	76.8 (23.8)	85.6 (15.5)
Mental component summary	52.4 (12.4)	53.7 (11.3)

^a^Medications: Beta-blocker: carvedilol; Angiotensin-converting enzyme inhibitors: enalapril and captopril; Angiotensin receptor blockers: losartan; Diuretics: furosemide and hydrochlorothiazide; Aldosterone antagonist: spironolactone; Anticoagulants: warfarin.

*CCC* chronic Chagas cardiomyopathy, *LVEF* left ventricular ejection fraction, *VO*_*2*_* peak* oxygen intake at peak exercise, *BMI* body mass index, *ACEI* angiotensin-converting enzyme inhibitors, *ARB* angiotensin receptor blockers, *SF-36* Medical Outcomes Study 36-Item Short-form of Health Survey, *QoL* quality of life.

### Effect of physical exercise training on quality of life

**Figure 2:**
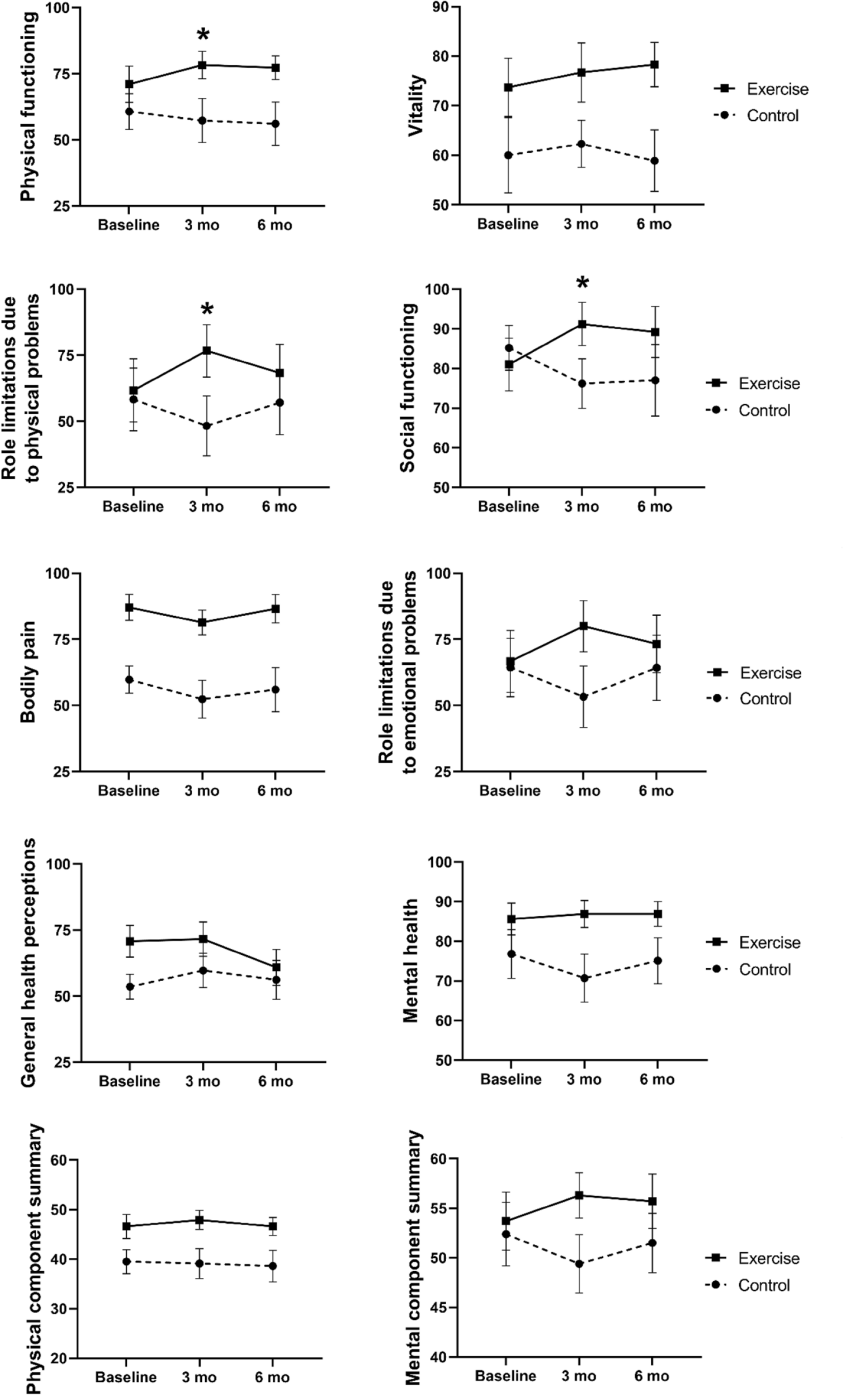
Estimated mean changes from baseline for quality of life.

The effects of physical exercise during the follow-up are depicted in Table 2, while Fig. 2 illustrates the crude trajectories for QoL scales during the follow-up in each group.

**Table 2:** Crude means (standard deviation) and adjusted beta values for QoL at 3 and 6- month of follow-up.

SF-36 QoL scales	3 months Control n = 15 (100%) Exercise n = 15 (100%)			6 months Control n = 14 (93.3%) Exercise n = 15 (100%)		
	Mean (sd)	*β*	p^a^	Mean (sd)	*β*	p^a^
Physical functioning						
Control	57.3 (32.1)	+ 10.7	**0.02**	56.1 (30.6)	+ 13.6	0.08
Exercise	78.3 (20.1)			77.3 (17.4)		
Role limitations due to physical problems						
Control	48.3 (43.8)	+ 25.0	**0.01**	57.1 (45.4)	+ 2.9	0.85
Exercise	76.7 (38.3)			68.3 (41.7)		
Bodily pain						
Control	52.4 (27.5)	+ 4.3	0.62	56.0 (31.3)	+ 7.2	0.48
Exercise	81.4 (18.1)			86.6 (20.9)		
General health perceptions						
Control	59.7 (25.2)	− 5.3	0.40	56.2 (27.5)	− 11.0	0.20
Exercise	71.6 (25.2)			60.9 (26.3)		
Physical component summary						
Control	39.1 (11.7)	+ 1.7	0.47	38.6 (11.9)	+ 0.6	0.84
Exercise	47.9 (7.4)			46.6 (7.1)		
Vitality						
Control	62.3 (18.4)	+ 0.7	0.13	58.9 (23.1)	+ 7.6	0.88
Exercise	76.7 (23.2)			78.3 (17.4)		
Social functioning						
Control	76.2 (24.1)	+ 19.2	** < 0.01**	77.0 (33.7)	+ 16.7	0.14
Exercise	91.2 (21.1)			89.2 (24.9)		
Role limitations due to emotional problems						
Control	53.3 (45.1)	+ 24.4	0.09	64.3 (46.2)	+ 10.4	0.59
Exercise	80.0 (37.4)			73.3 (42.2)		
Mental health						
Control	70.7 (23.3)	+ 7.5	0.09	75.1 (21.6)	+ 7.2	0.23
Exercise	86.9 (13.2)			86.9 (12.1)		
Mental component summary						
Control	49.4 (11.4)	+ 5.6	0.06	51.5 (11.2)	+ 4.7	0.28
Exercise	56.3 (8.8)			55.7 (10.6)		

^a^Linear mixed models including the interaction between time, group, and time x group adjusted for baseline values.

*SF-36* Medical Outcomes Study 36-Item Short-form of Health Survey, *QoL* quality of life.

Estimates in [bold] are statistically significant

There were significant improvements in physical functioning (β =  + 10.7; *p* = 0.02), role limitations due to physical problems (β =  + 25.0; *p* = 0.01) and social functioning (β =  + 19.2; *p* < 0.01) scales during the first three months in the exercise group compared to the control group. However, no significant differences were observed between groups after six months of follow-up.

## Discussion

The main findings of the present study consisted of short-term improvements in some physical and mental aspects of QoL. These results are in accordance with previous studies that suggested a beneficial influence of exercise training on QoL of patients with CCC^16,18^. In addition, the relatively high compliance with the exercise protocol used in the present study in comparison to previous studies in the literature suggests that CR is a feasible strategy and should be encouraged as a coadjuvant in the treatment of CCC^36–38^.

The influence of exercise training on QoL among patients with other cardiovascular diseases has been previously investigated in the literature. A meta-analysis of randomized controlled trials conducted by Dallas et al. included 5,786 patients with HF and showed significant improvements on physical and mental QoL scales following exercise training (− 0.82, 95% CI − 1.02 to − 0.62; *p* = 0.00001), regardless of the instrument used to measure QoL^14^. On the other hand, Quittan et al. used the SF-36 questionnaire to compare the effects of three months of aerobic exercise training (intervention group) or usual daily living activities (control group) in the QoL of HF patients. Similar to our results, they found significant improvements in physical functioning and role limitations due to physical problems scales^39^.

Considering patients with CCC, the studies conducted so far presented conflicting results. Mediano et al. concluded that an 8-month exercise-based CR program improved the physical scales of QoL in a sample of patients with severe CCC^18^. In opposition, the study conducted by Lima et al. found unchanged physical QoL aspects following exercise training^16^. This surprising lack of improvement observed in the latter study may be explained by the fact that 63% of the patients did not present any kind of functional limitations (NYHA class I), with the remaining 37% presenting only mild limitations (NYHA class II)^16^. On the other hand, a greater percentage of patients included in our study referred some degree of functional limitations, representing a population that can benefit more from the improvements in functional capacity promoted by physical exercise training^20^.

Social functioning scale usually reflects the impact of physical health or emotional issues on social activities^25^. Interestingly, it was the only mental scale that presented a significant improvement following exercise training, although role limitations due to emotional problems, mental health, and MCS reached borderline p-values after three months of follow-up (*p* < 0.10). This result may be related to improvements on functional capacity due to exercise training^20^, which could enable patients to engage in a greater number of social activities. In addition, participation in a CR program, with regular social support from the CR team and other patients, appears to be beneficial for the patient's social functioning by itself^40^. Our results are partially in line with those observed by Lima et al., that also found improvements in QoL mental scales (vitality, role limitations due to emotional problems, and mental health, but not in social functioning)^16^. Conversely, Mediano et al. found no positive influence of an 8-month exercise-training program on any QoL mental scales in patients with severe CCC^18^. Studies including patients with heart diseases from other etiologies did not show significant effects of physical exercise on the social functioning scale^21,39^. Patients with CCC may experience distinct psychosocial challenges, including stigma, anxiety, and depression, which could affect their social functioning differently than patients with other more prevalent heart diseases. Sociocultural differences among patient populations with different heart diseases may also influence their perceptions of and responses to CR programs, potentially impacting their social functioning outcomes^36,37,41–43^.

The short-term improvements (3 months) on QoL obtained in the exercise training group in comparison to controls were not sustained up to the end of the study follow-up (6 months), except for the borderline change on physical functioning scale after six months of follow-up (*p* = 0.08). This unexpected result can be partially explained by the potential influence of the nutritional and pharmaceutical interventions offered to the control group throughout the study period, which may have positively impacted their QoL, as previously demonstrated^44^. In addition, the multidisciplinary care provided during each physical exercise session, which allowed a close observation of potential clinical decompensation that were treated immediately, may have influenced the result^45^. Moreover, untrained individuals tend to present optimistic expectations when starting a physical exercise program. Thus, the level of interest may be higher in the first three months, decreasing later, which was reflected in the reduction in the perception of QoL at the end of the six months of follow-up^46^.

The present study has limitations and strengths. First, considering that exercise training is a behavioral intervention, masking the study participants as well as the CR team was not possible. The greater frequency of interactions between participants in the exercise training group and CR health professionals may have influenced the differences observed in QoL outcomes between the groups. However, it is important to acknowledge that in a clinical trial focusing on physical exercise within a cardiac rehabilitation context, mitigating such influences is always challenging. Nevertheless, participants from both groups attended monthly appointments with their cardiologist, in addition to receiving identical nutritional and pharmaceutical counseling during the study. These efforts were made to ensure parity in non-exercise aspects of care. Secondly, despite SF-36 is the most widely used instrument to evaluate QoL, this instrument is not validated for use in CD population, which may result in a less accurate assessment of QoL and increased risk of nondifferential error. Moreover, the SF-36 questionnaire may not evaluate specific cognitive features associated with physical activity, such as self-efficacy, which has been demonstrated to affect QoL changes related to physical activity^47,48^. The lack of evaluation of total physical activity levels during the study (besides attendance rates to training sessions) may also be ackowledged as a limitation, making difficult the identification of participants that changed their physical activity habits during the study, especially in the control group, which may have driven our results to the null hypothesis. Besides that, the sample was composed of patients regularly followed in a national reference center for treatment of infectious diseases, which may limit the external validity. The sample size was calculated based only on primary outcome, which prevented us from conducting subgroup analysis. However, the study design with strict patient´s follow-up and the relatively long-term duration of the study are potential strengths.

To conclude, the main finding of the present study is that exercise-based CR provided short-term improvements in the physical and mental aspects of QoL of patients with CCC. These results are of great clinical meaning since CR is a simple and low-cost tool to improve patients' QoL, especially in endemic areas. Future studies examining the dose–response relationship between exercise and QoL and potential influence of different lifestyle intervention strategies (isolated or combined) on long-term QoL responses are necessary to support clinical recommendations for patients with CCC.

## Data Availability

The datasets used and/or analysed during the current study are available from the corresponding author on reasonable request.
